# Keratoconus-like tomographic changes in a case of recurrent interstitial keratitis

**DOI:** 10.1186/s12348-018-0146-7

**Published:** 2018-03-07

**Authors:** Marie-Sophie Hanet, Annette Zimpfer, Sabine Lepper, Berthold Seitz

**Affiliations:** 1 Department of Ophthalmology, Saarland University Medical Center, 66424 Homburg, Saar Germany; 2 Institute of Pathology, Saarland University Medical Center, 66424 Homburg, Saar Germany

**Keywords:** Keratoconus, Interstitial keratitis, Inflammation

## Abstract

**Background:**

The purpose of this report was to describe a single case of recurrent interstitial keratitis in a young patient leading to keratoconus-like corneal changes.

**Results:**

Over the 2 years follow-up, the patient developed clinical signs of keratoconus with Vogt’s striae, Fleischer Ring and paracentral stromal thinning in the affected eye only. The tomographic measurements revealed a rapid reduction of corneal thickness from 581 to 303 μm and an irregular steepening of the pathological cornea. True net corneal power increased from 42.8 to 48.8 dioptres (D) and maximal power of the anterior surface from 44.9 to 66.9 D. Best-corrected visual acuity (BCVA) decreased from 20/20 to 20/200. A penetrating excimer laser keratoplasty was performed to restore vision and corneal stability.

**Conclusion:**

Keratoconus-like changes can occur in young patients with recurrent interstitial keratitis and request corneal transplantation.

## Findings

### Introduction

Keratoconus is a degenerative disorder of the cornea associated with corneal thinning and ectasia that leads to refractive errors and impaired vision. It is mostly bilateral, typically affects adolescents or young adults and may progress until the third or fourth decade of life, when it usually arrests [1].

The pathophysiology of keratoconus is complex and still remains unclear.

Recent studies revealed the probable influence of inflammatory mechanisms in the pathogenesis of keratoconus and emphasises the importance of controlling inflammation to prevent progression of the disease [2–4].

Infectious keratitis can cause topographic changes resulting in visual impairment. This phenomenon was recently investigated, and tomographic measurements of post-infected corneas revealed different patterns of corneal aberrations including protrusion keratoconus-like patterns [5, 6].

In support of these considerations, we report the case of a young patient with clinical manifestations of corneal inflammation, who developed strictly unilateral keratoconus-like changes in the affected eye.

### Case report

A 27-year-old Caucasian man was referred to our center with complaints of persistent blurred vision on the left eye. He was treated with corticoid eye drops and systemic acyclovir tablets for the past 2 months on the suspicion of herpetic keratitis. No other ophthalmological pathology or systemic disease was documented.

**Fig. 1:**
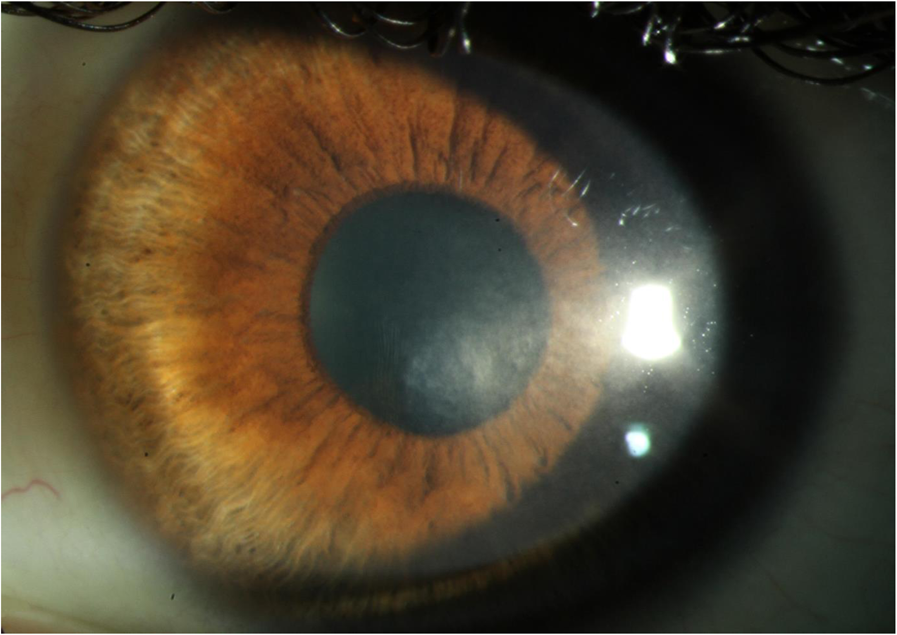
Slit-lamp photograph of the left eye a few months after initial presentation. The inferonasal part of the cornea showed a hazy opacification of the deep stroma

On initial presentation, he had a best-corrected visual acuity (BCVA) of 20/20 in both eyes. His refraction was − 3.25 sphere/− 0.50 cylinder at 180° in the right eye and − 3.50 sphere/− 1.00 cylinder at 175° in the left eye. The slit lamp examination revealed a hazy opacification of the deep stroma in the inferior nasal part of the left cornea with endothelial deposits (Fig. 1). Tomographic measurements (Oculus-Pentacam HR) revealed maximal anterior, posterior and mean total keratometry values from 44.9, − 6.1 D and 42.8 D respectively, and a minimal corneal thickness of 581 μm on the left eye. Optical coherence tomography (Casia SS-100, Tomey) showed a deep stromal hyperreflexity in the corneal center of the left eye. The endothelial cell count with specular microscopy (Tomey EM-3000) was minimally reduced to 2408 cells/mm^2^ in the left eye in comparison with the right eye (2525 cells/mm^2^). Blood analyses showed resolved VZV and HSV infections (enzyme-linked immunosorbent assay, ELISA), resolved CMV infection (electrochemiluminescence immunoassay, ECLIA) and hepatitis B immunity (ECLIA). No active infection (incl. borrelia) or systemic inflammation could be found; thorax radiology was without pathology.

Based on the clinical diagnosis of non-necrotising stromal keratitis of presumed herpetic origin, we treated the patient with corticoid eye drops, ganciclovir eye gel and acyclovir tablets. During the 2-year follow-up of the patient, relapses occurred with reduction of the therapy, which required regular increases of the frequency of topical therapy application or temporary use of systemic steroid.

**Fig. 2:**
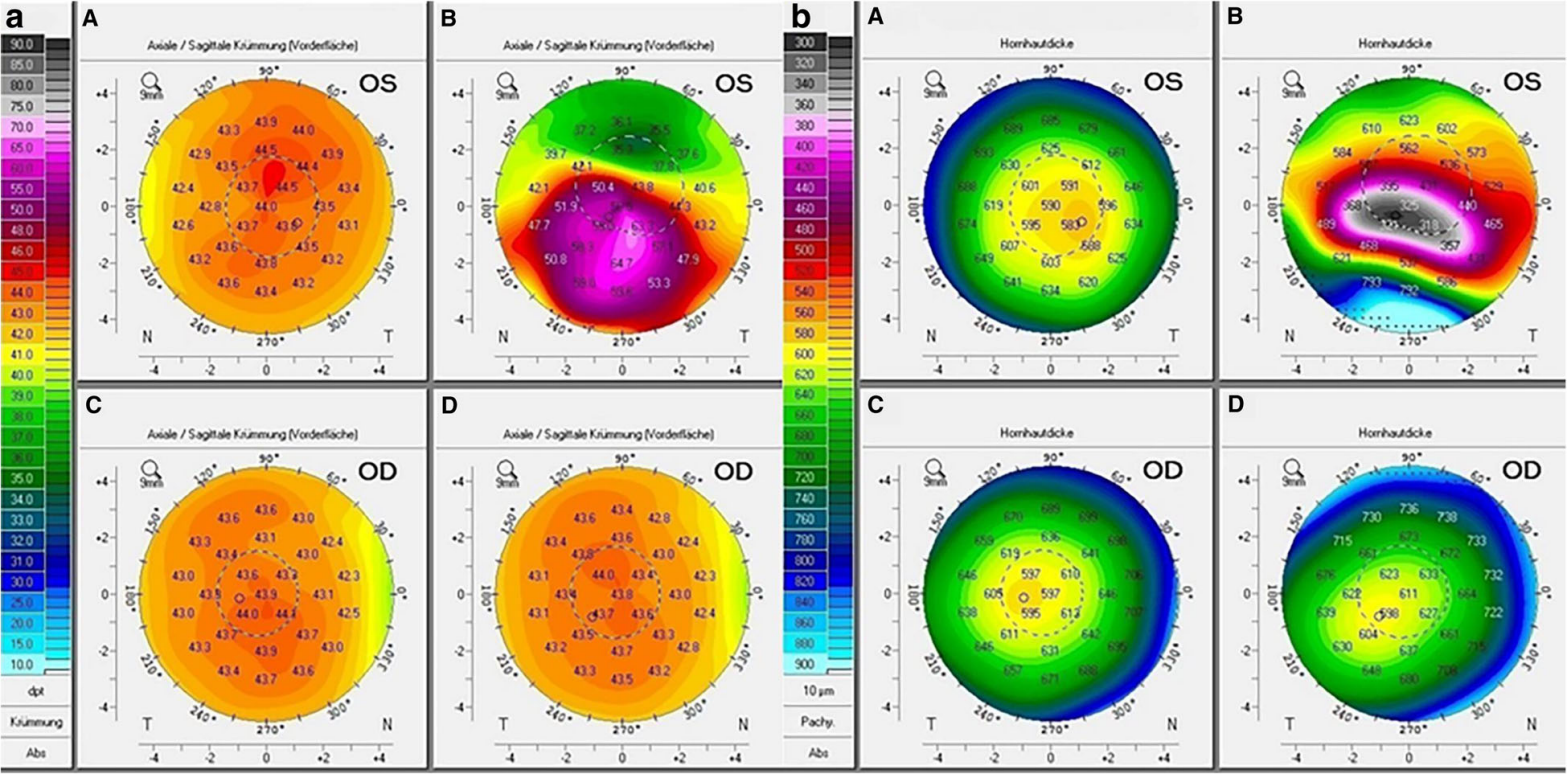
Pentacam tomography showing **a** maximal power of the anterior surface of the left eye (OS) increased between day of presentation (A, 44.9D) and last control before corneal transplantation (B, 66.9D). **b** Corneal thickness of the left eye decreased between day of presentation (A, 581 μm) and last control before corneal transplantation (B, 303 μm). No changes are noticed on the right eye between day of presentation (**c**) and last control before corneal transplantation (**d**). *N* nasal, *T* temporal

**Fig. 3:**
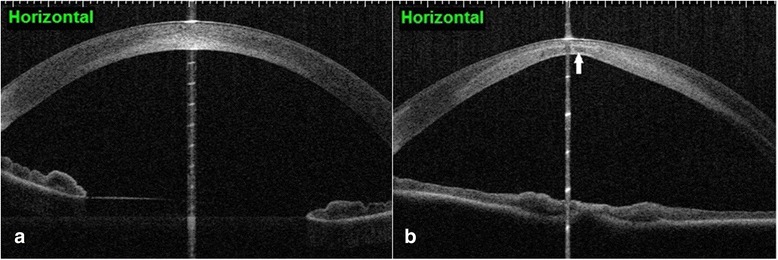
Optical coherence tomography of the left eye at day of presentation (**a**) and at last control before keratoplasty (**b**) showing a cone-shaped bulge of the cornea with corneal thinning and increase of stromal hyper-reflexivity in the posterior part (arrow)

**Fig. 4:**
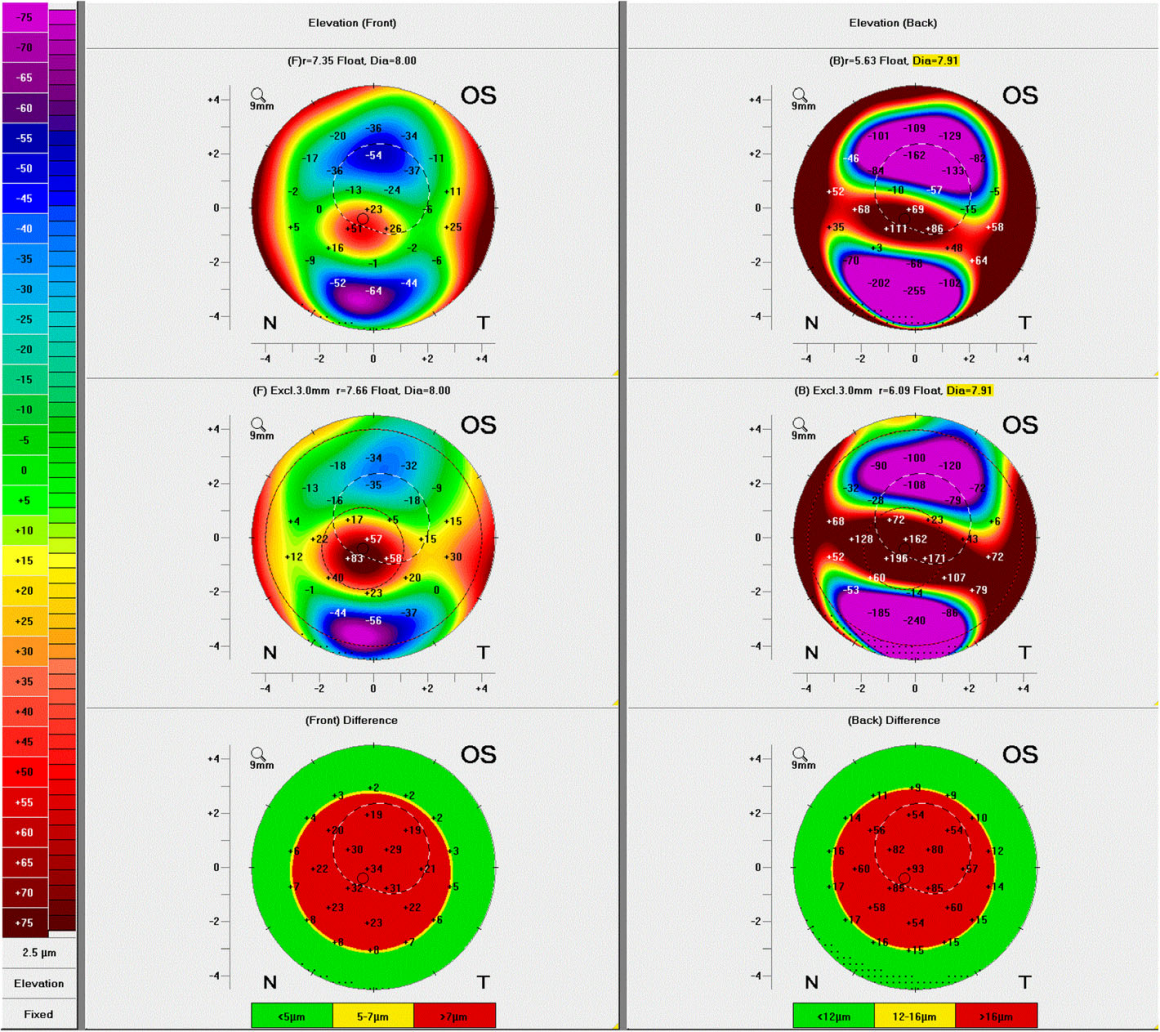
Elevation maps of the anterior (left) and posterior surface (right) of the left eye before corneal transplantation

Despite regular controls and therapy adaptation, clinical examinations showed keratoconus-like changes with Vogt’s striae, incomplete Fleischer Ring and signs of paracentral stroma thinning on the left eye, starting 1 year after onset of the disease. Two years after presentation, BCVA of the affected eye was reduced to 20/200; maximal anterior, posterior and true net powers had increased to 66.9, − 8.1 and 48.8 D respectively; minimal corneal thickness decreased to 303 μm; and optical coherence tomography showed a cone-shaped bulge of the paracentral cornea with increased stromal hyper-reflexivity (Figs. 2, 3 and 4, Table 1).

**Table 1:** Evolution of the left eye

		Month 0	Month 4	Month 11	Month 16	Month 19	Month 22
BCVA		20/20	20/20	20/20	20/100	20/200	20/200
Pachymetry at the thinnest point (μm)		581	n.a.	511	427	360	303
Power of the anterior surface (dpt)	K1	43.3	43.2	42.7	44.8	44.2	49.0
	K2	44.4	44.4	43.2	46.1	47.0	52.3
	Kmax	44.9	44.8	45.5	49.4	59.3	66.9
Power of the posterior surface (dpt)	K1	− 6.0	− 5.5	− 6.4	− 6.7	− 8.0	− 7.8
	K2	− 6.2	− 5.8	− 5.9	− 6.3	− 7.3	− 8.4
	Kmax	− 6.1	− 5.6	− 6.1	− 6.5	− 7.6	− 8.1
True net power (dpt)	Km	42.8	43.2	41.7	44.2	44.1	48.8

*K1* power of the steep meridian, *K2* power of the flat meridian, *Kmax* maximal corneal power, *Km* mean keratometry measure, *n*.*a*. not available

An excimer laser supported penetrating corneal transplantation was carried out at this point on the left eye.

**Fig. 5:**
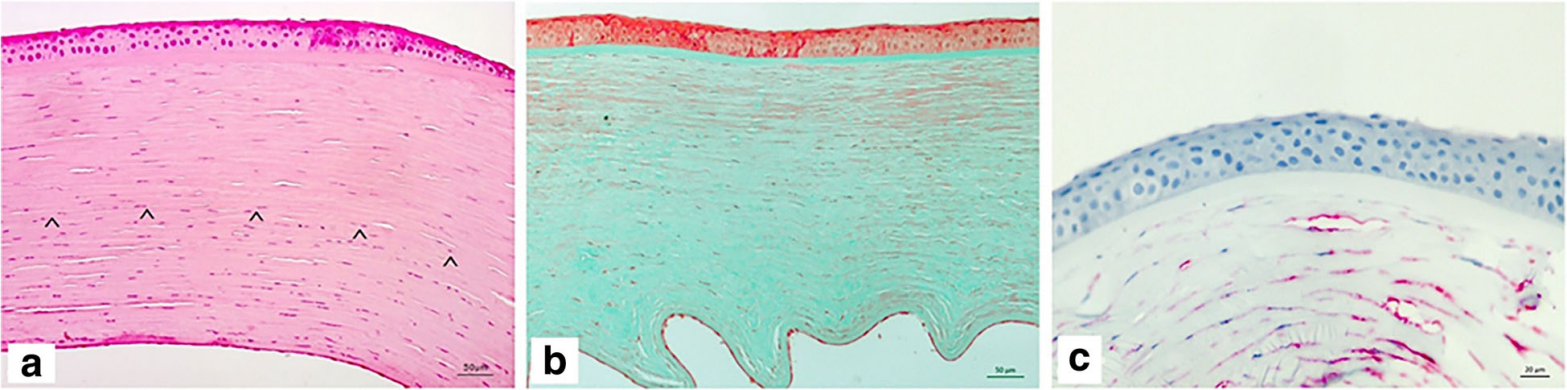
**a**–**c** Chronic (stromal) interstitial keratitis. **a** The cornea is edematous and slightly infiltrated by lymphocytes and monocytes in the posterior part (arrowheads) (haematoxylin-eosin, × 20). **b** Masson-Goldner stain shows increase of irregular collagen bundles in the middle and deep stromal parts (× 20). **c** Myofibroblastic activation of stromal fibrocytes are highlighted by CD34 immunohistochemistry (× 20)

Pathological analyses of the excised cornea showed a chronic (stromal) interstitial keratitis with stromal scarring, edema and myofibroblastic activation of stromal cell (Fig. 5a–c). In special stains (Congo red, Grocott-Gomori’s, PAS, Gram) and in situ hybridization, the presence of fungus, borrelia, cytomegalie virus, herpes simplex virus (type I and II) or EBV were excluded. Three months after transplantation, the patient recovered a BCVA of 20/32 on the left eye and no recurrence was observed to date.

## Discussion

Originally, keratoconus was presented as a non-inflammatory disorder affecting primarily young men, usually bilateral and leading to visual impairment. Although the pathogenesis of keratoconus remains poorly understood, multiple studies revealed the importance of inflammation in the progression of the disease and a recent consensus was to consider keratoconus an inflammatory disease [3, 4]. Accordingly, novel therapeutic approaches aiming to prevent keratoconus progression through reduction of inflammation were evaluated. The topical application of immune-modulator cyclosporine A demonstrated encouraging results in a small amount of keratoconus patients [2].

Eye rubbing, atopy or contact lens wear were also revealed as risk factors in clinical and experimental studies, suggesting the role of environmental aspects [7–9]. As eye rubbing showed to be associated with increased levels of proinflammatory mediators in tears, its influence on keratoconus may be related to pro-inflammatory effect [7]. Keratoconus has also been associated with metabolic disorders of the urea cycle [10] and thyroid gland dysfunction [11]. Multiple studies support the influence of genetic components in the pathogenesis of the disease [9, 12].

In recent studies, the effect of infectious keratitis on corneal topography was investigated [5, 6]. The topographic changes due to infectious keratitis seem to depend on the location of the inflammation and the healing response of the host. Although asymmetric patterns appeared to be the most common, protrusion patterns were also revealed [5]. The pathogenesis of these changes has not been elucidated yet, but the first patterns may be associated to stiffening of the inflamed cornea due to a predominance of scarring process, whereas the second, keratoconus-like patterns would arise through stromal melting. Optical aberrations caused by topographic changes of the cornea were revealed, as well as corneal opacification, to be responsible for visual impairment following infectious keratitis [5].

Our patient had no systemic disease or familial history of keratoconus. He developed rapid keratoconus-like corneal changes on the eye with recurrent inflammatory manifestations but no clinical signs of keratoconus in the other eye. This clinical case may support the essential role of inflammation in the development and progression of keratoconus-like ecstasies. Although the cause remains unclear, the rapidity of the progression of keratoconus in this case could be related to the age of the patient, which correlates with the usual onset of the disease.

This case demonstrates that keratitis may lead to keratoconus-like corneal changes and supports the hypothesis of a causative link between recurrent inflammatory process and the development of keratoconus. It provides additional arguments towards the use of stronger anti-inflammatory medications or immunosuppressive drugs such as cyclosporine in similar cases to prevent corneal ectasia and corneal transplantation.
